# Volumetric Passive Acoustic Mapping and Cavitation Detection of Nanobubbles under Low-Frequency Insonation

**DOI:** 10.1021/acsmaterialsau.4c00064

**Published:** 2024-10-29

**Authors:** Hila Shinar, Tali Ilovitsh

**Affiliations:** 1 Department of Biomedical Engineering, Tel Aviv University, Tel Aviv 6997801, Israel; 2 The Sagol School of Neuroscience, Tel Aviv University, Tel Aviv 6997801, Israel

**Keywords:** microbubbles, nanobubbles, cavitation monitoring, passive cavitation detection, passive acoustic mapping, low-frequency ultrasound

## Abstract

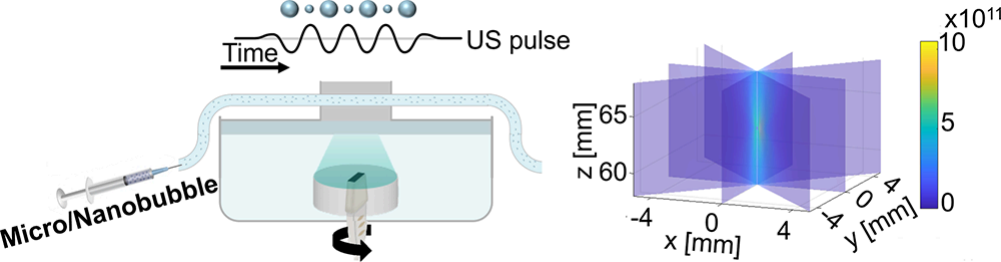

Gas bubbles, commonly used in medical ultrasound (US), witness advancements with nanobubbles (NB), providing improved capabilities over microbubbles (MB). NBs offer enhanced penetration into capillaries and the ability to extravasate into tumors following systemic injection, alongside prolonged circulation and persistent acoustic contrast. Low-frequency insonation (<1 MHz) with NBs holds great potential in inducing significant bioeffects, making the monitoring of their acoustic response critical to achieving therapeutic goals. We introduce a US-guided focused US system comprising a one-dimensional (1D) motorized rotating imaging transducer positioned within a low-frequency therapeutic transducer (center frequencies of 105 and 200 kHz), facilitating precise monitoring of NB cavitation activity in three-dimensional (3D) and comparison with MBs. Passive cavitation detection (PCD) revealed frequency-dependent responses, with NBs exhibiting significantly higher stable and inertial cavitation doses compared to MBs of the same gas volume when excited at a center frequency of 105 kHz and peak negative pressures ranging from 100 to 350 kPa. At 200 kHz, MBs showed higher cavitation doses than NBs. PCD showed that 105 kHz enhanced both NBs’ and MBs’ oscillations compared to 200 kHz. The system was further used for 3D passive acoustic mapping (PAM) to provide spatial resolution alongside PCD monitoring. Two-dimensional PAM was captured for each rotation angle and used to generate a complete 3D PAM reconstruction. Experimental results obtained from a tube phantom demonstrated consistent contrast PAM full-width half-maximum (FWHM) as a function of rotation angle, with similar FWHM between MBs and NBs. Frequency-selective PAM maps distinguished between stable and inertial cavitation via the harmonic, ultraharmonic and broadband content, offering insights into cavitation dynamics. These findings highlight NBs’ superior performance at lower frequencies. The developed 3D-PAM technique with a 1D transducer presents a promising technology for real-time, noninvasive monitoring of cavitation-based US therapies.

## 1 Introduction

The integration of microbubbles (MBs) and nanobubbles (NBs) as contrast agents, in conjunction with ultrasound (US), has significantly advanced the fields of medical imaging and therapy. MBs, gas-filled spheres typically ranging from 1 to 7 μm in diameter, are widely used in US imaging and diagnostics.^1^ Their high acoustic impedance mismatch with biological tissues and their compressible nature make them ideal US contrast agents.^2^ Moreover, the potential of MBs extends far beyond diagnostic applications. They hold significant promise for various therapeutic applications, including gene therapy, drug delivery, tissue ablation, and blood–brain barrier (BBB) opening.^1−5^ The use of low-frequency insonation (<1 MHz) has shown potential in enhancing the therapeutic effects of MBs and NBs by inducing high-amplitude oscillations at lower pressures compared to traditional MHz-range US.^1,4,6−13^ Low-frequency US also creates a larger focal zone, enabling the treatment of larger tissue volumes.^3,13,14^ Additionally, low-frequency US provides advantages in terms of reduced trans-skull aberrations.^14,15^ In the context of US-mediated brain therapies, low frequency is critically important as it allows for deeper penetration through the skull and lower attenuation passing through tissue.^3,16−21^ Our previous research demonstrated that using low frequencies enhances the MB vibrational response and enables safe BBB opening.^4^One limitation of MBs is their micrometer diameter, which restricts their utility to intravascular applications as they cannot extravasate from blood vessels into surrounding tissues. Additionally, their size limits their maneuvering in small blood vessels, potentially reducing their penetration into capillaries.^22−26^ To address these challenges, a new class of smaller US contrast agents, NBs, was developed. Similar to MBs, NBs are gas-filled spheres, but with diameters of less than 1 μm.^1,2,27−30^ NB’s unique properties hold promise for various biomedical applications. Notably, their smaller size facilitates deeper tissue penetration and enhances extravasation from blood vessels into targeted cells.^2,27,30^ In addition, NBs have prolonged circulation time and persistent acoustic contrast,^31−33^ which makes them useful contrast agents. These characteristics make NBs especially appealing for therapeutic applications.^3,34^ Nevertheless, our understanding of the dynamics of NBs remains incomplete due to their small diameter, which presents challenges for optical imaging.^35,36^ Alternatively, acoustical response characterization could be employed; however, their small size may result in weaker signals that require a sensitive system for capturing the acoustic response.^2^ In this article, we aim to address this gap by characterizing the acoustic response of NBs under low-frequency US excitation using a US-guided focused US system. Furthermore, we conduct a comparative analysis between NBs and MBs to shed light on the distinct advantages offered by each type of contrast agent for US-based therapies.

The key to the therapeutic potential of MBs and NBs lies in the cavitation phenomenon.^37,38^ Cavitation occurs upon excitation of the bubbles by US waves and can manifest in two primary forms: stable and inertial cavitation.^1,39−42^ Stable cavitation involves sustained bubble oscillations, characterized by repetitive expansion and contraction that release relatively low levels of energy. Stable cavitation finds significant applications in diagnostic and therapeutic procedures, particularly for enhancing drug delivery through controlled mechanical effects. It also holds promise for procedures involving BBB opening.^38^ Stable cavitation is crucially important for BBB disruption as it promotes temporary and localized disruption, allowing for the passage of therapeutic agents without causing significant damage to surrounding tissue.^3,8,34^ In contrast, inertial cavitation involves the rapid and forceful collapse of bubbles, generating intense shock waves, microjets, and localized pressure changes. Compared to stable cavitation, inertial cavitation releases higher energy levels, harnessed in medical treatments like histotripsy for tissue ablation and targeted disruption of pathological conditions.^3,38^ Distinguishing between these two cavitation forms is crucial for tailoring US therapies, optimizing outcomes, and minimizing side effects.^8,39,40,43^

Passive cavitation detection (PCD) is a widely used technique for monitoring the cavitation activity. PCD captures the acoustic emissions produced by the natural cavitation events of the bubbles. By recording and analyzing these passive acoustic signals, PCD offers insights into the occurrence, characteristics, and intensity of cavitation.^3,38,40,44−46^ This allows for real-time assessment without disruption of the biological or experimental system. While this noninvasive approach is essential for monitoring and optimizing cavitation-based medical interventions, it primarily provides spectral information rather than spatial cavitation localization. To overcome this limitation, passive acoustic mapping (PAM) emerged as a critical technique.^46−53^ PAM is specifically designed for the spatial characterization of cavitation activity. This involves extracting relevant information from acquired signals to construct spatial maps, enabling the visualization of cavitation distribution and characteristics within the study area.^38,54^ Traditional PAM methods utilize a 1D transducer to generate a space-dependent representation of cavitation through a 2D cavitation map.^38,54,55^ Since a 1D array is used to characterize the acoustic response generated by a 3D anatomic volume, 2D PAM is inherently incomplete. Full 3D PAM requires the use of a 2D transducer.^54,56^ Yet, the limited availability and high cost of 2D transducers pose a significant challenge,^54^ highlighting the need for a modality that enables 3D cavitation mapping using a readily available and cost-effective technology. To address this need, we introduce a new technique that enhances volumetric PAM by incorporating a 1D motorized rotating imaging transducer positioned within a therapeutic transducer. By leveraging precise rotation coordinates with 2D PAM, the generation of a complete 3D PAM from 2D slices is achieved, providing valuable spatial information about the cavitational behavior of MBs and NBs under low-frequency US excitation.

The aim of the paper is 2-fold. On the one hand, the setup that was built, which includes an imaging transducer rotating in a controlled manner within a therapeutic transducer and allows for the extension of bubble cavitation characterization methods, specifically PAM, into 3D. This expansion can shed further light on cavitation processes and characterize the spatial behavior during treatment, aiding in both treatment localization and 3D monitoring. This adds a new layer of information that was previously unavailable with 1D transducers. This setup was used for the direct comparison of the behavior of MBs versus NBs, which is the second objective of the paper. Currently, the use of NBs is gaining momentum in imaging and therapeutic applications, and there is a growing need for sensitive methods to characterize their behavior and compare them to those of MBs. This is essential for understanding their advantages over MBs and optimizing their use for the appropriate applications.

## 2 Methods

### 2.1 Microbubble Preparation

The MBs were composed of a perfluorobutane (C_4_F_10_) gas core encapsulated in a lipid shell as described in ref (57). The lipid shell consisted of 1,2-distearoyl-*sn*-glycero-3-phosphocholine (DSPC) and 1,2-distearoyl-*sn*-glycero-3-phosphoethanolamine-*N*-[methoxy(polyethylene glycol)-2000] (ammonium salt) (DSPE-mPEG2000) (Sigma Aldrich, St. Louis, MO, USA). The lipids were mixed in a ratio of 90:10 mol/mol and dissolved into propylene glycol by heating and sonicating at 62 °C. A mixture of glycerol (Gly, Acros Organics) and phosphate buffer solution (PBS, pH 7.4, 02-023-1A, Biological Industries Israel, Beit-HaEmek, Israel), which was preheated to 62 °C, was added to the lipid solution. The combined mixture was sonicated for 10 min at room temperature. The final lipid concentration was 2.5 mg/mL. The solution was aliquoted into vials with a liquid volume of 1 mL. The air was manually removed from the vials using a syringe and was replaced with perfluorobutane gas (C_4_F_10_). The vials were sealed and stored at 4 °C. Prior to their use, the MBs were activated by mechanical shaking for 45 s in a Vialmix shaker (Bristol-Myers Squibb Medical Imaging Inc., N. Billerica, MA). Purification and size selection of the suspension were then achieved through the differential centrifugation process.^4^ The MB suspension underwent centrifuging at 300 relative centrifugal force (RCF) for 10 min and collection of the white bubble cake. Next, MBs larger than 10 μm were eliminated by centrifuging at 16 RCF for 1 min, followed by centrifugation at 45 RCF for 1 min and collection of the turbid suspension. MBs smaller than 1 μm were eliminated by three times centrifugation at 300 RCF for 3 min and collection of the white bubble cake. The size and concentration of the MBs were measured twice with a particle counter system (AccuSizer FX-Nano, Particle Sizing Systems, Entegris, MA, USA). The size distribution and concentration varied by less than 10% between the two measurements. The MB solution was used within 3 h of preparation. The fabricated MBs had an average diameter of 1.24 ± 0.73 μm (Figure S1). Prior to the injection of the MB solution to the phantom, the solution was diluted to a concentration of 1 × 10^8^ MBs/mL.

### 2.2 Nanobubble Preparation

The NBs were composed of an octafluoropropane (C_3_F_8_) gas core encapsulated in a lipid shell as describe in ref (57). The lipid shell was comprised of 1,2-dibe-henoyl-*sn*-glycero-3-phosphocholine (C22), 1,2-dipalmitoyl-*sn*-glycero-3-phosphate (DPPA), 1,2-dipalmitoyl-*sn*-glycero-3-phos-phoethanolamine (DPPE), and 1,2-distearoyl-*sn*-glycero-3-phos-phoethanolamine-*N*-[methoxy(polyethylene glycol)-2000] (ammonium salt) (DSPE-mPEG 2000) (Sigma-Aldrich). The lipids were mixed in a C22:DPPA:DPPE:DSPE-mPEG2000 ratio of 18.8:4.2:8.1:1 mol/mol/mol/mol and dissolved into propylene glycol solution by heating and sonicating at 80 °C. A mixture of glycerol and phosphate buffer solution (PBS, pH 7.4), which was preheated to 80 °C, was added to the lipid solution and sonicated for 10 min at room temperature. The resulting lipid concentration was 10 mg/mL. The solution was aliquoted into vials with a liquid volume of 1 mL. The air was manually removed from the vials and replaced with octafluoropropane (C_3_F_8_). The vials were sealed and stored at 4 °C. Prior to their use, the NBs were activated by mechanical shaking for 45 s in a Vialmix shaker. The NBs were isolated from the mixture of foam and MBs by centrifugation at 50 RCF for 5 min. The NBs were then manually withdrawn from a fixed distance of 5 mm from the bottom of the vial using a 21G needle. The size and concentration of the NBs were measured twice with a particle counter system (AccuSizer FX-Nano, Particle Sizing Systems, Entegris, MA, USA). The size distribution and concentration varied by less than 10% between the two measurements. The NB solution was used within 3 h of preparation. The fabricated NBs had an average diameter of 0.16 ± 0.02 μm (Figure S1). Prior to the injection of the NB solution into the phantom, the solution was diluted to a concentration of 5 × 10^10^ NBs/mL.

### 2.3 Ultrasound Setup

**Figure 1:**
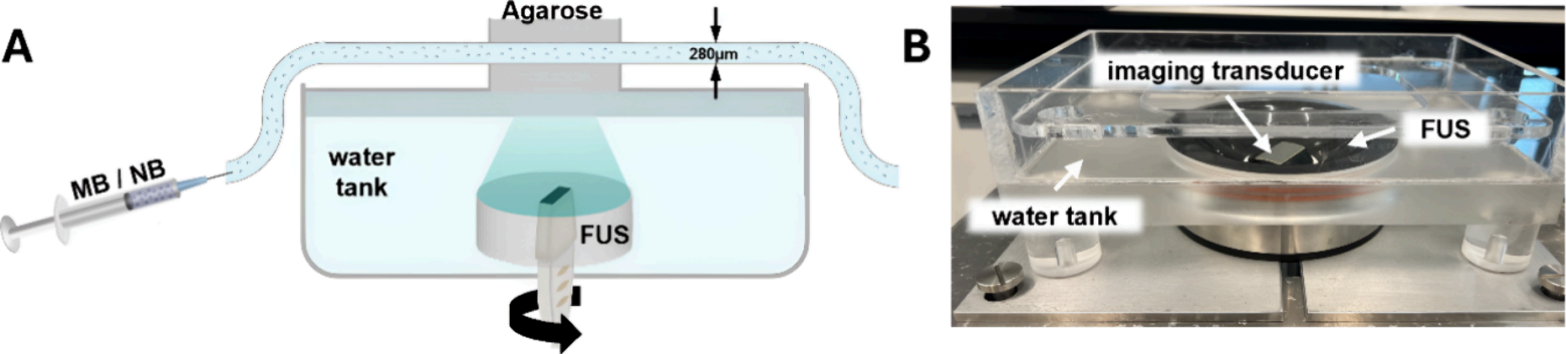
Experimental setup. (A) Illustration of the experimental setup. A syringe pump was used to inject the bubbles into a tube embedded in an agarose phantom. The phantom was placed at the focal spot of the USgFUS system. (B) USgFUS system composed of two concentric transducers: a rotating array imaging transducer and a concave focused US therapeutic transducer.

PCD and PAM experiments were conducted by using a US guided focused US (USgFUS) setup. The setup consisted of two concentric US transducers: a therapeutic transducer and an imaging transducer (Figure 1). The therapeutic transducer (H149, Sonic Concepts, Sonic Concepts, Bothell, WA, USA) was a single-element concave focusing transducer with a focal distance of 63 mm, which could support two center frequencies: 105 and 200 kHz. The therapeutic transducer was connected to a matching network corresponding to the chosen center frequency. The imaging transducer (IP104, Sonic Concepts, Sonic Concepts, Bothell, WA, USA) was a 1D linear array transducer with 128 elements and a 3.47 MHz center frequency. The imaging transducer was placed at a central opening in the middle of the therapeutic transducer so that the two transducers were coaligned and concentric. The focal spot of the therapeutic transducer was always contained within the imaging array’s field of view. The imaging transducer was assembled into a motorized rotary (RTY-IP100, Sonic Concepts), composed of a rotary motor and a gear system. The motor enabled a 360° rotation of the imaging transducer relative to the fixed therapeutic transducer. The rotation angle, speed, and acceleration were controlled via MATLAB. The integrated transducer complex was placed at the bottom of a water tank. Distilled water was degassed in gallon containers under house vacuum for at least 24 h prior to each experiment. Before each experiment, the water tank was filled with degassed deionized water, and an agarose phantom was placed at the focal spot of the therapeutic transducer. The transmission and recording of the US signal were operated by an US programmable system (Vantage 256, Verasonics Inc., Redmond, WA, USA) and controlled via MATLAB. Upon receiving a trigger from the software, the therapeutic transducer generated a US pulse, and the imaging transducer recorded the acoustic emissions. A function generator (AFG1022, Tektronix, Beaverton, OR, USA) was used to generate the desired signal, which was then amplified with a radio frequency (RF) amplifier (2100L, E&I, Rochester, NY, USA). The transducers were calibrated in advance using a needle hydrophone (NH0200, Precision Acoustics, UK). A calibration of the hydrophone for frequencies in the frequency range of 100–1000 kHz was performed by the company at the UK National Physical Laboratory.

### 2.4 Agarose Phantom Fabrication

During PCD and PAM experiments, the bubbles were injected into a thin polyethylene (PE) tube embedded in a tissue-mimicking agarose phantom. The PE tube (BTPE-10, Instech laboratories Inc., Plymouth meeting, PA, USA) featured an inner diameter of 280 μm and served as a blood vessel mimicking phantom. The tube was inserted into a custom-made laser-cut mold. The mold was of a rectangular cuboid shape and had a size of 65 mm × 30 mm × 25 mm (Figure S2). The mold contained four rectangular faces that were assembled together into a cuboid. In the center of the two parallel faces, a hole was drilled to fit the tube. After the mold was assembled and the tube was inserted, an agarose solution was poured into the mold to embed the tube. The solution was prepared by dissolving 1.5% agarose powder (Alfa Caesar, MA, USA) in deionized water, followed by microwaving to eliminate air bubbles. The superheated solution was poured into the mold and allowed to cool down at room temperature until fully solidified. Then, each face of the mold was removed to extract the final phantom that was situated on the top of the water tank of the transducers (Figure 1A). To minimize standing waves and reduce reflections, an 8 mm rubber layer was attached to the top surface of the phantom and coupled using a US gel. In each experiment, a solution containing either MBs, NBs, or degassed PBS was injected into the PE tube at a constant rate of 0.5 mL/min using a syringe pump (GenieTouch, Kent Scientific, Torrington, USA). The injected solution concentration was 1 × 10^8^ MBs/mL for the MBs and 5 × 10^10^ NBs/mL for NBs. These concentrations were chosen to match the gas volumes of the MB and NB solutions.

### 2.5 Passive Cavitation Detection

The USgFUS setup was used to quantify the cavitation activity of MBs and NBs within the tube phantom following US excitation. The bubbles were placed in a syringe and injected at a constant flow rate of 0.5 mL/min into a 280 μm tube by using a syringe pump. The tube was embedded in an agarose phantom, which was positioned on the top of the water tank at the focus of the therapeutic transducer. Upon initiating a custom MATLAB program, a trigger signal was sent to the function generator, prompting the therapeutic transducer to transmit a 100 cycle burst pulse at the chosen frequency (105 or 200 kHz) and a specified peak negative pressure (PNP) ranging from 100 to 350 kPa. The US parameters were selected based on optimization performed in a previous paper.^8^ The chosen frequencies were low because, as shown in this former study, the use of low frequencies enhances bubble cavitation intensity. The specific setup supported two frequencies (200 and 105 kHz), allowing for a direct comparison between them. Simultaneously, the rotating imaging array captured the acoustic emissions of the bubbles, which were saved as radio frequency (RF) data. In the PCD experiments, the imaging array remained stationary at a fixed angle of 0° with respect to the tube. The sampling frequency was selected as four times the center frequency of the imaging transducer (e.g., 13.88 MHz). Six independent measurements were performed for each set of parameters.

The first 400 μs of the recorded RF data underwent the following processing procedure. Zero-padding with 512 elements was applied to mitigate wraparound errors during the frequency spectrum calculation. Then, a fast Fourier transform (FFT) was performed to obtain the corresponding frequency spectrum. Element-wise averaging of the FFT was carried out over the 128 elements of the array. Then, the stable cavitation dose (SCD) and the inertial cavitation dose (ICD) were calculated. Harmonics and ultraharmonics were used as indicative of stable cavitation and broadband signal served as indicative of inertial cavitation.^58−61^ Thereby, for SCD, the harmonic and ultraharmonic frequencies emitted by the bubbles were isolated using a comb band-pass filter. Nine consecutive harmonic components (e.g., 2*f*_0_, 3*f*_0_, etc.) and nine consecutive ultraharmonic components (e.g., 2.5*f*_0_, 3.5*f*_0_, etc.) were targeted, each analyzed within a 2.6 kHz rectangular window centered at its corresponding frequency. The root-mean-square (RMS) of each frequency band was calculated, and the SCD was determined as the sum of all of the RMS values across all bands. An average SCD was calculated based on the six independent measurements. To account for background noise unrelated to cavitation, the averaged control SCD (of a PBS solution) was subtracted from the averaged bubbles’ SCD. For ICD, the broadband signal was extracted by emitting the harmonic and ultraharmonic frequency bands that were used for SCD. The remaining frequency bands were used for ICD calculation. The RMS value of each frequency band was calculated, and the ICD was obtained by summing the RMS values of all bands. An average SCD was calculated based on the six independent measurements. Finally, the average control ICD was subtracted from the average bubbles’ ICD.

### 2.6 Three-Dimensional Passive Acoustic Mapping

The framework used for PCD was used for PAM as well. To achieve three-dimensional spatial characterization, the imaging transducer was programmed to rotate and change its orientation in relation to the bubbles. Initially, the imaging transducer was aligned parallel to the tube. During the experiment, fresh bubbles were injected into the tube through a syringe pump at a constant flow rate of 0.5 mL/min. The bubbles were excited by a continuous 200 kHz US signal. A MATLAB program was utilized to rotate the imaging transducer in 5° increments. Following each angle increment, the imaging transducer paused and recorded the RF signal. The cycle of rotation and recording was repeated until the transducer reached a final position of 180° relative to its initial orientation. Six independent recordings were performed for each set of parameters. The recorded RF data was used then processed using the angular-spectrum PAM (AS-PAM) algorithm^62^ to reconstruct 2D and 3D PAM images.

The AS-PAM algorithm is a powerful tool for reconstructing spatial maps of the cavitation activity intensity. The AS-PAM algorithm exploits the angular spectrum of the acoustic field, measured at a specific plane, to numerically estimate the acoustic field in other parallel planes. Consider a scenario where the acoustic signal is captured by a 1D transducer positioned at plane *z*_0_ along the *x*-axis. The intensity of the acoustic field at a parallel plane, *z*_1_, along the *x*-axis, can be calculated as:^62^
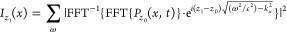
1where *P*_z0_ is the recorded acoustic signal as a function of time (*t*), ω is the angular frequency, *K_x_* is the wavenumber in the *x*-direction, *c* is the speed of sound in the working medium, FFT represents the Fast Fourier Transform, and ∑ indicates summation over all angular frequencies. This formula performs a 1D back-projection of the signal using a transfer function. It is important to note that the formula assumes a homogeneous medium and neglects the possibility of multiple scattering events. These limitations are crucial to consider when interpreting the reconstructed maps. To reconstruct a 2D spatial map using the AS-PAM algorithm, the formula must be iteratively applied across a range of axial locations (*z*_1_) within the region of interest. This allows for the estimation of the acoustic field intensity at various depths within the sample, ultimately building a comprehensive 2D representation of the cavitation distribution.

### 2.7 Cavitation Map Reconstruction

2D cavitation maps were reconstructed by the following procedure. First, the recorded RF data underwent high-pass filtering at 1.5*f*_0_ to eliminate backscattered signals irrelevant to bubble interactions, followed by zero-padding with 512 elements to mitigate wraparound errors during the frequency spectrum calculation. Then, a fast Fourier transform (FFT) was performed on each RF measurement to obtain its corresponding frequency spectrum. Equation 1 was used to reconstruct the 2D cavitation map from the frequency spectrum. The field of view (FOV) for each map was set to 40 mm × 27.94 mm in the axial (*z*) and lateral (*x*) directions, respectively. The lateral FOV was dictated by the size of the imaging transducer, while the axial FOV ensured complete capture of the focal region. The pixel size of the map was 0.22 mm (lateral) × 0.21 mm (axial). To enhance the signal-to-noise ratio and reduce random variations, the 2D maps from all six independent measurements were averaged to generate the mean 2D PAM. Background noise unrelated to bubble cavitation was removed by subtracting control measurements (PBS only, with no bubbles) from the bubble maps. The full width at half-maximum (FWHM) of each 2D map was extracted along both the axial and lateral directions to quantify the spatial extent of the cavitation activity.

After the reconstruction of the 2D maps of the entire frequency spectrum, a similar process was used to reconstruct 2D frequency-selective PAM. In addition to the 2D map, which consisted of the entire frequency spectrum, three types of frequency-selective maps were composed for each set of parameters: (1) contained only the harmonic content, (2) contained only the ultraharmonic content, and (3) contained the remaining broadband signal. The harmonics and ultraharmonics were isolated using a comb band-pass filter. Nine consecutive harmonic components (e.g., 2*f*_0_, 3*f*_0_, etc.) were targeted, as well as nine consecutive ultraharmonics (e.g., 2.5*f*_0_, 3.5*f*_0_, etc.), and the broadband noise. Each harmonic and ultraharmonic were analyzed within a 2.6 kHz rectangular window centered at its corresponding frequency.

3D map reconstruction employed a set of 2D cavitation maps (of the entire frequency spectrum) associated with different rotational angles of the imaging transducer. A 3D voxel mesh was preallocated in memory as the output volume, and the 2D images were spatially registered according to their known rotation angle. Finally, interpolation was performed using MATLAB’s “scatteredInterpolant” function to increase the image pixel density. The final 3D map had a 0.1 mm × 0.1 mm × 0.1 mm voxel size and an FOV of 5 mm (sagittal) × 5 mm (coronal) × 10 mm (axial).

## 3 Results

**Figure 2:**
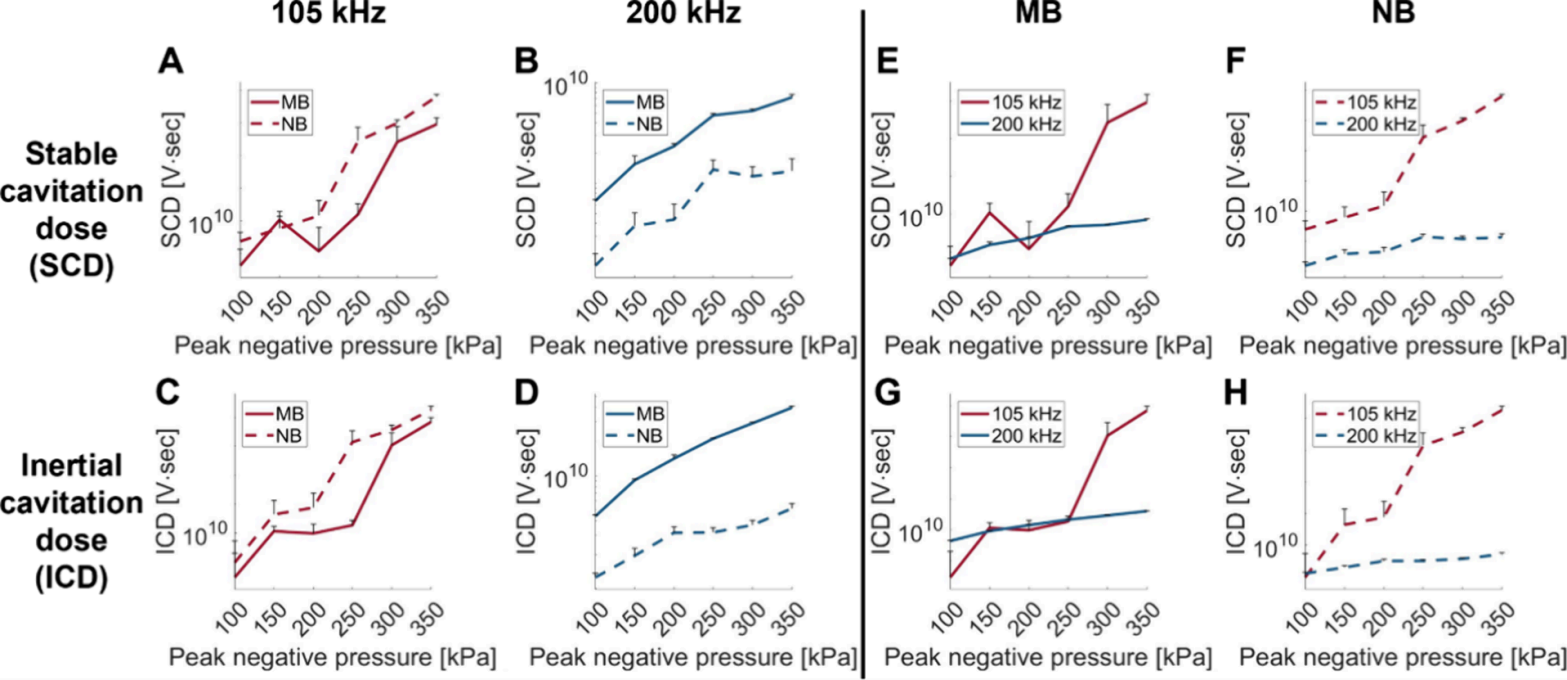
Passive cavitation detection of MB and NB as a function of applied PNP. SCD and ICD results were obtained using different experimental parameters. Left: a comparison between MB and NB excited at a center frequency of (A, C) 105 and (B, D) 200 kHz. Right: a comparison between the two excitation frequencies (105 and 200 kHz), using (E, G) MB and (F, H) NB. The error bars represent the standard deviation for six independent measurements.

The characterization of MBs and NBs was conducted by using multiple techniques. Microscopy images were used to visualize the MBs (Figure S1A), but due to the diffraction limit, this technique could not be used to image NBs. Therefore, transmission electron microscopy was employed for NB imaging (Figure S1B). To measure the size distribution and concentration of the bubbles, the Accusizer FX Nano system was utilized with typical results shown in Figure S1C,D. PCD was employed to characterize the cavitation activity of MBs and NBs within a 280 μm tube. The bubbles were exposed to US excitation at 105 and 200 kHz with varying PNPs from 100 to 350 kPa. The recorded data was subsequently analyzed to compute the SCD and the ICD of the bubbles. Both SCD and ICD exhibited a clear dependence on the PNP and US frequency (Figure 2). In all experiments involving both MBs and NBs at 105 and 200 kHz, increasing the PNP resulted in progressively higher SCDs and ICDs. At an excitation frequency of 105 kHz, NBs consistently induced higher SCDs and ICDs compared with MBs at PNPs exceeding 200 kPa (Figure 2A,C). However, at 200 kHz, an opposing trend was observed. MBs induced higher SCD and ICDs compared to NBs, at all measured PNPs (Figure 2B,D). These findings are consistent with our previous observations on NBs and MBs.^9^

**Figure 3:**
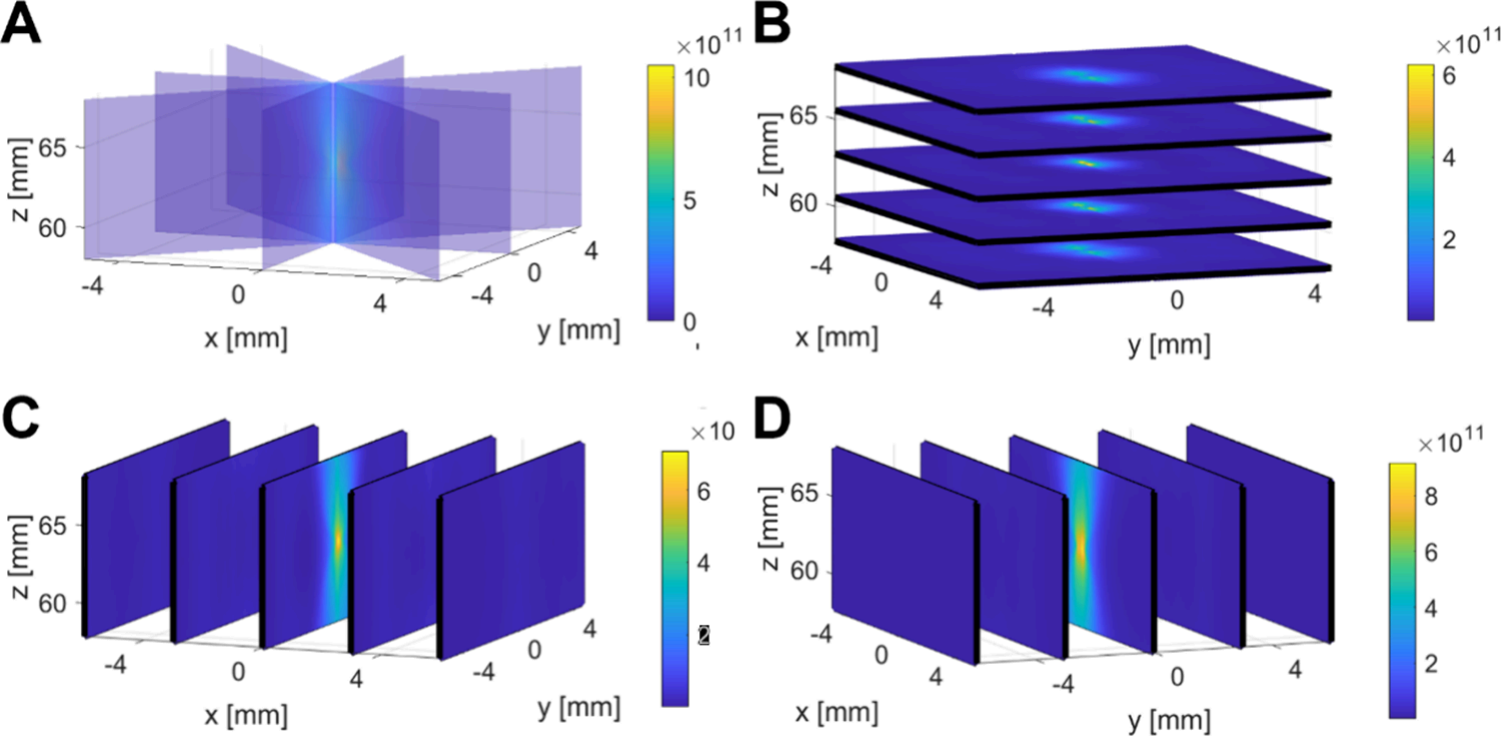
Three-dimensional passive acoustic maps of nanobubbles at 200 kHz and a PNP of 300 kPa. 3D PAM displayed in different sections. (A) As a function of the rotation angle. (B) Parallel to the *X*–*Y* plane, (C) *Y*–*Z* plane, and (D) *X*–*Z* plane. All colorbars are in A.U.

Next, 3D-PAM was used to provide spatiotemporal information along with the cavitation activity of the bubbles. MB and NBs were excited at 200 kHz and a PNP of 300 kPa, and the rotating 1D imaging transducer was used to record their emission at rotation angles ranging from 0 to 180° at 5° increments. The recorded data was used to reconstruct acoustic maps, representing the cavitation intensity of the MBs and NBs in 2D. The set of 2D PAM images was then stacked based on the known angle at which each frame was acquired, forming a 3D PAM. For visualization, the 3D PAM was displayed in four configurations: at intersecting planes (of 45° angle increments) (Figure 3A) and at different sections, including axial, sagittal, and coronal views (Figure 3B–D). A localized activity of the NBs was observed near the intersection of the different planes, with a peak cavitation intensity of 5.868 × 10^11^ A.U.

**Figure 4:**
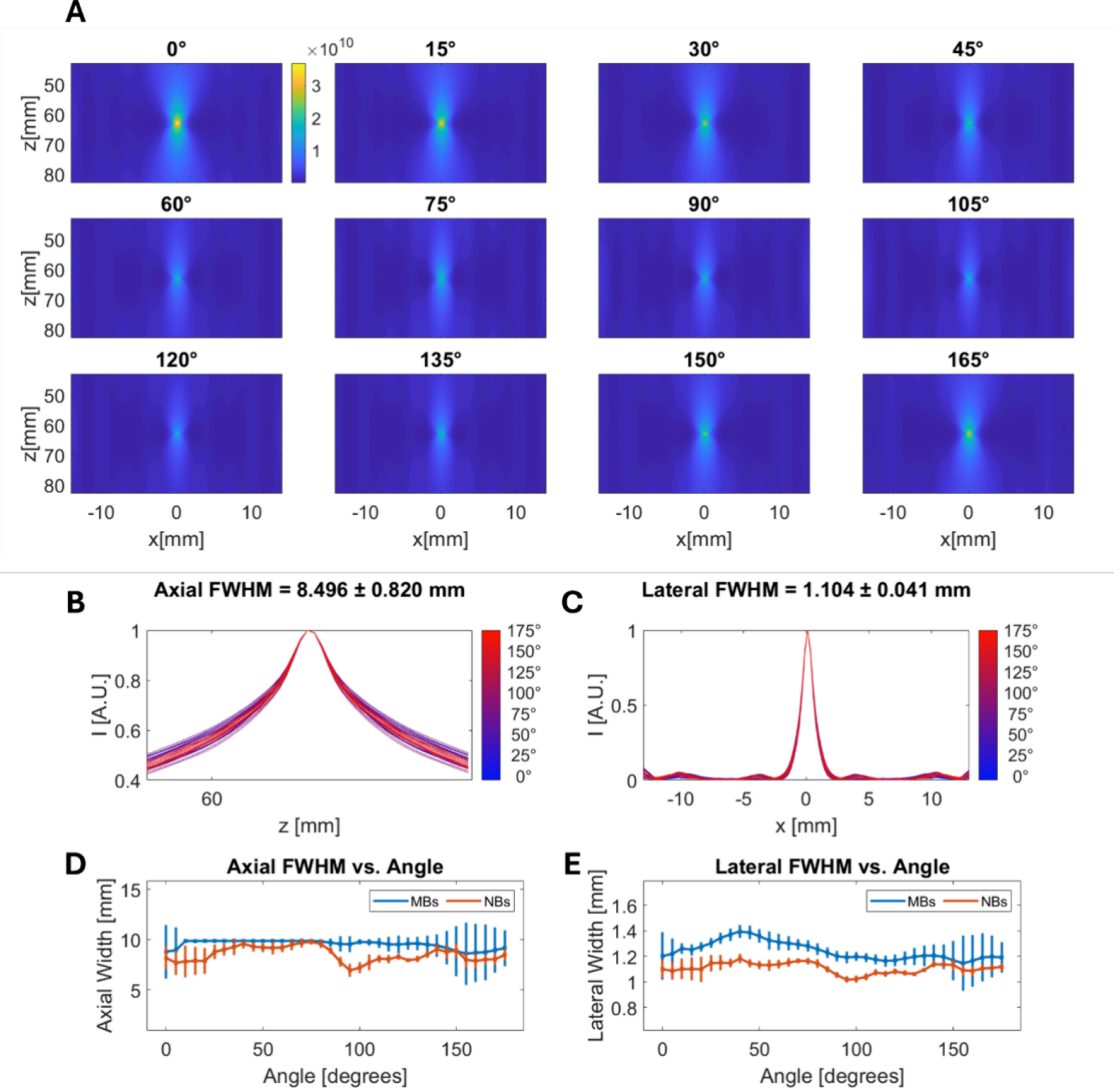
Angle-dependent 3D passive acoustic mapping of nanobubbles. (A) Passive acoustic maps of the NBs at different angles. The NBs were excited at 200 kHz and a PNP of 300 kPa. Axes and color bars are common to all of the subfigures. The color bar is in A.U. (B, C) Intensity profiles of the PAM at the axial and lateral directions. (D, E) The full width at half-maximum (FWHM) at the axial and lateral directions as function of transducer angle. The error bars represent the standard deviation for six independent measurements.

Then, the angle dependency of the cavitation activity was investigated. 2D acoustic maps at different angles were reconstructed for MB and NB (Figure S3 and Figure 4A, respectively). These maps revealed a consistent cavitation distribution in space, with variations primarily in focal spot intensity. The intensity profiles of the NBs along axial and lateral directions (Figure 4B,C) exhibit mean FWHM of 8.5 ± 0.8 and 1.1 ± 0.1 mm, respectively, while the MBs exhibited mean axial and lateral FWHM of 10.8 ± 1.5 and 1.2 ± 0.09 mm. This demonstrated the anticipated localization of cavitation at the focal spot of the therapeutic transducer. FWHM showed no dependency on the rotation angle in the axial and lateral directions (Figure 4D,E).

**Figure 5:**
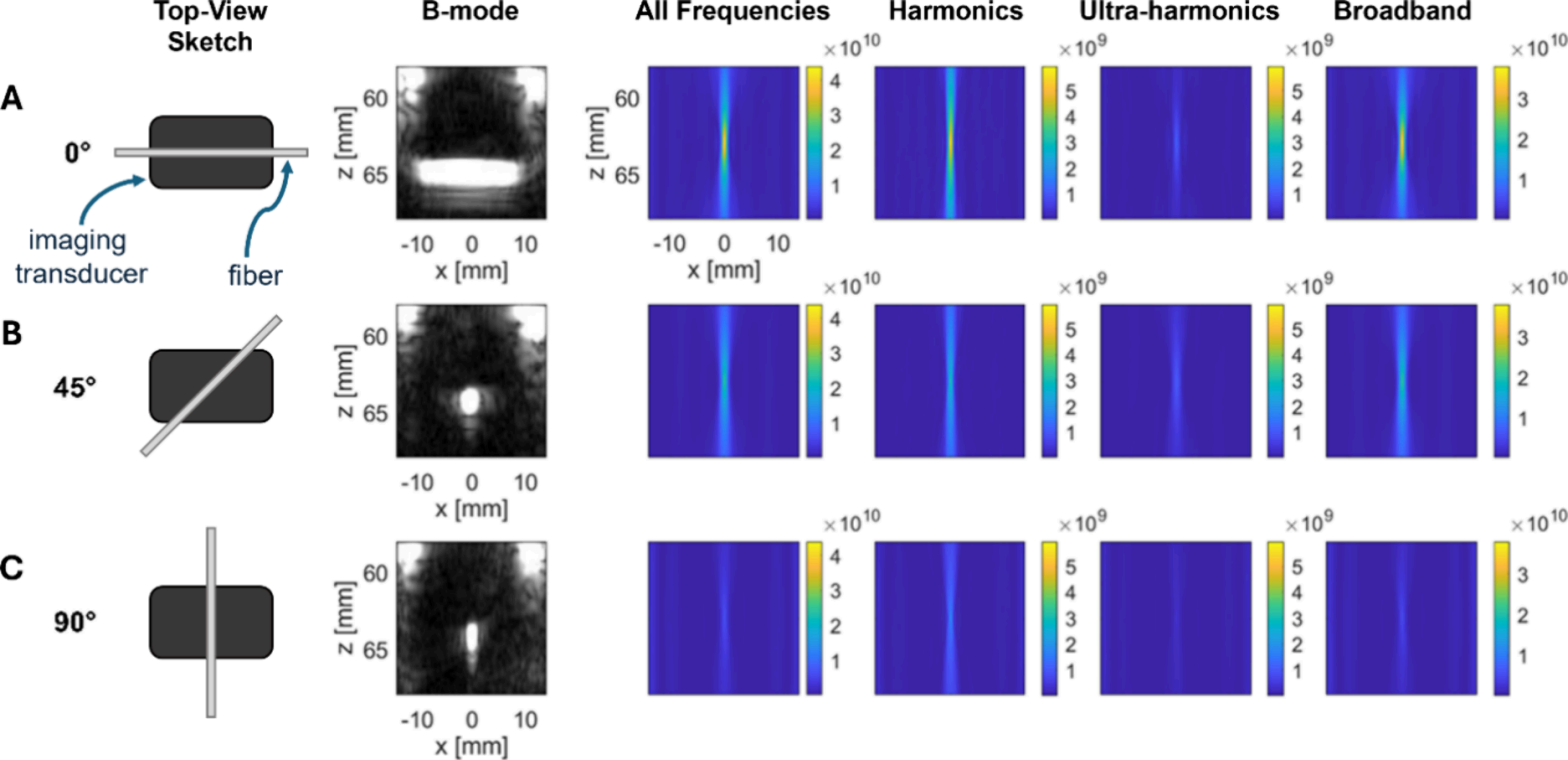
Passive acoustic mapping of nanobubbles at individual angles of 0, 45, and 90°. NBs were excited at 200 kHz and a PNP of 300 kPa. The cavitation activity of the NBs was assessed at (A) 0°, (B) 45°, and (C) 90°. For each angle, the PAM images are presented next to the B-mode image of the phantom and a top-view sketch demonstrating the orientation of the fiber relative to the imaging transducer. PAM is reconstructed using the entire spectrum (“All Frequencies”), only the harmonics, only the ultraharmonics, or using the broadband signal. All color bars are in A.U.

To provide a higher resolution investigation of the angular dependence, three specific angles of 0, 45, and 90° with respect to the tube were chosen for the MB and NB (Figure S4 and Figure 5, respectively). For each angle, the B-mode US images present the 2D cross section of the tube. Next, the PAM algorithm was executed and the frequency spectra at each angle were extracted. The spectra contain peaks at the harmonics and ultraharmonics of the center frequency. The intensity of the peaks is influenced by the cavitation intensity, as well as by the order of the harmonic (or ultraharmonic). On the one hand, peaks at higher frequencies are diminished as they require greater amount of nonlinearity of the bubbles. On the other hand, higher harmonics fall in the bandwidth of the imaging transducer (1–5 MHz) and thus tend to be more pronounced, as observed here. Received frequency components were separated to present either the entire frequency bandwidth, the harmonic content, the ultraharmonic content, or the broadband content. For both MB and NB, all three angles exhibited a clear signal at all four types of cavitation maps, as supported by the PCD experiments.

## 4 Discussion

US is widely recognized as one of the most versatile medical modalities, offering numerous advantages. It is noninvasive, easy to operate, cost-effective, and capable of penetrating deep into the human body with minimal risk of damage. The introduction of MBs and NBs has further expanded the US utility, with some applications already available and many more currently in different stages of research and clinical trials.^1,2^ In this study, we present a technique for mapping cavitation activity in three dimensions by using a 1D imaging array. The technique is based on the well-established PAM technique and utilizes the AS-PAM algorithm. Using this method, we investigated the interaction of MBs and NBs with low-frequency US, aiming to enhance our understanding of their acoustic characteristics. We complemented our advanced setup with PCD and 2D-PAM for a more comprehensive understanding of the MB and NB acoustic behavior. While PAM was previously employed to map nanoemulsions,^63^ nanocups,^64^ and nanodroplets,^65^ to the best of our knowledge, this study represents the first application of PAM for NB characterization.

PCD was employed for assessing the amount of cavitation in the bubbles. Two metrics were used for this purpose, SCD and ICD, where SCD refers to stable cavitation and ICD refers to inertial cavitation. In BBB opening procedures, the use of appropriate cavitation is essential for the success of the treatment and the prevention of unwanted damage. Here, we evaluated these metrics for different peak negative pressures and for MBs and NBs to examine the sensitivity of the method and characterize the behavior of the bubbles across each parameter. This characterization will enable the correct selection of operating parameters during US treatments and, therefore, constitutes a necessary step before conducting experiments.

PCD analysis revealed that both MBs and NBs exhibited higher cavitation doses at the lower measured US frequency (105 kHz) compared with the higher frequency (200 kHz), indicating more efficient energy transfer at lower frequencies. This trend remained consistent across all experiments involving both MBs and NBs at these frequencies. However, even with matched gas volumes to the MBs, NBs demonstrated a more pronounced acoustic response to the US signal at 105 kHz. At 200 kHz, on the other hand, MBs produced greater cavitation doses compared with the NBs. These contrasting trends suggest that the acoustic response of MBs and NBs is highly dependent on the US frequency. Further research is needed to gain a broader understanding of this effect. This also emphasizes the necessity for a comprehensive investigation into the dynamic properties of NBs, which have not been thoroughly explored to date. Moreover, the high acoustic response of NBs at 105 kHz highlight their unique potential. Combining this enhanced acoustic response at specific frequencies with other advantages, such as deeper body penetration, enhanced cellular extravasation, and potentially improved biocompatibility, positions NBs as promising candidates for diverse therapeutic interventions. Furthermore, the distinct acoustic responses of MBs and NBs under low-frequency US excitation emphasize the importance of selecting the appropriate contrast agent for specific applications, while carefully considering the purpose of the treatment and the desired bioeffects.

PAM provided a detailed spatial characterization of cavitation activity for both NBs and MBs. The reconstructed maps were captured at various angles, peak negative pressures, and frequencies, offering clear visualizations of the cavitation localization and distribution. The data acquisition involved transmitting a therapeutic pulse and then receiving the returning signal by the imaging transducer, and this process was repeated for each transmission angle. It is possible that in this manner, we measured signals from different bubbles during each acquisition. The situation likely mimics what will occur in vivo due to the flow of bubbles within the blood vessels. Our results showed consistent cavitation distribution across PAM of different angles; variations were mainly in focal spot intensity rather than in the FWHM or the intensity pattern. This aligns with the circular geometry of the therapeutic transducer. In addition to angle-dependent investigations, we reconstructed frequency-specific PAM maps. For each measurement, four types of maps were generated: one consisting of the entire frequency spectrum and the other two containing only the harmonic, ultraharmonic, or broadband content. These maps not only provided insights into cavitation intensity and its spatial distribution but also revealed the nature of cavitation: stable vs inertial cavitation. The signal in the frequency-selective maps was consistent with the corresponding frequency spectrum. These findings highlight the potential benefits of constructing cavitation maps from specific frequency components. Importantly, this study introduced an advanced experimental setup that enables accurate 3D cavitation mapping using a single rotating 1D transducer. A similar approach was previously employed for B-mode and color-doppler imaging.^66^ The use of the 1D array approach surpasses techniques that utilize 2D arrays for reconstruction of 3D-PAM, by providing a more feasible and cost-effective alternative. This technique offers a noninvasive solution for various applications requiring bubble cavitation monitoring and shows promise for BBB opening procedures, where precise control over cavitation type (stable vs inertial) is essential for minimizing vascular damage.

This study has limitations related to both cavitation detection and the PAM technique itself. The cavitation detection technique struggles to differentiate between similar frequency spectra arising from distinct underlying phenomena. For example, harmonic components can originate from strong, localized pressure fluctuations within the medium rather than from stable cavitation events. Moreover, the AS-PAM algorithm assumes nonabsorbing, homogeneous media, and point-like receivers for the imaging array, potentially leading to inaccuracies in estimations of cavitation intensity or location. While some researchers have addressed these limitations,^37,53,62,67,68^ further efforts are required in this regard. Furthermore, PAM images exhibit limited spatial resolution, particularly in the axial direction. This constraint has been previously discussed in other studies^38,54,62,67,68^ and requires further exploration before practical implementation. The resolution is determined by the recorded acoustic emissions’ frequency content, the imaging array geometry, the experimental setup, and the reconstruction algorithm. Mitigating the axial resolution limitation could involve using larger imaging apertures, incorporating different spatial interpolation techniques, or employing super-resolution imaging methods. However, these approaches may entail increased hardware costs or reconstruction times, potentially impeding real-time monitoring and restricting the modality availability. The challenge of low axial resolution is particularly relevant for low-frequency US signals, as employed in this study. Here, we partially mitigated this limitation by capturing higher-order harmonics and ultraharmonics, thereby achieving resolution comparable to studies utilizing higher center frequency excitation.^38,53,54,67^ Consequently, more sensitive setups capable of recording higher harmonics could enhance the PAM resolution. In terms of temporal resolution, it should be noted that our technique requires rotation of the imaging array during the measurement. As a result, the temporal resolution is reduced compared with techniques that use 2D arrays, which are able to capture the entire 3D space simultaneously. This presents a limitation only for applications that require high temporal resolution. Another limitation of the study is the uncertainty in the measurement of the NB concentration due to the presence of liposomes in the suspension. The liposomes, which have a diameter of about 50–500 nm,^69^ might have been mistakenly detected as NBs by the measurement device, leading to an overestimation of the NB concentration. To account for the potential overestimation of NB concentration and achieve a comparable gas volume between NBs and MBs, a higher concentration of NBs should have been used in the experiment. This would have resulted in an increased signal from the NBs, further amplifying their advantage in terms of generating greater cavitation doses at 105 kHz. Additionally, the experiments were conducted in a nonsymmetric tube phantom, which might introduce variability in the angle-dependent analysis of PAM images. Lastly, this study utilized MB and NB formulations that have been shown to be effective for theranostic applications in our lab and by other research groups.^8,22,23,57,70,71^ Factors such as bubble formulation, size, shell composition, and gas type can all influence the bubble vibrational response.^72−74^ The method presented here serves as a platform for bubble characterization, enabling comparisons of various parameters that affect the acoustic response of bubbles. Subsequent work will be conducted in vivo to further test the performance of this method and explore its feasibility for real-time monitoring. Future work will also include analysis of the subharmonic components of the center frequency, which could provide additional information about the dynamics of the bubbles.

In conclusion, this study achieved two key objectives. First, it demonstrates the enhanced acoustic response of NBs under low-frequency excitation. This could facilitate applications such as deep tissue targeting and BBB disruption. Second, the new technique of 3D-PAM utilizing a 1D transducer offers detailed cavitation information. This technique is low cost and more accessible compared to techniques that utilize 2D arrays and holds promise for monitoring US therapies in real time and noninvasively.

## Data Availability

The datasets generated during and/or analyzed during the current study are available from the corresponding author upon reasonable request.

## Supporting Information Available

Supplementary figures including MB and NB characterization, phantom fabrication process, and 3D-PAM of MB, including mapping of different angles and frequency-selective mapping (PDF)

## Notes

During the preparation of this work, the authors used ChatGPT-3.5 and Gemini in order to improve readability and language of the manuscript. After using this tool/service, the author(s) reviewed and edited the content as needed and take full responsibility for the content of the publication.
