# Supplementary material for: Volumetric Passive Acoustic Mapping and Cavitation Detection of Nanobubbles under Low-Frequency Insonation
Source: ACS Mater Au. 2024 Oct 29;5(1):159–69. doi: 10.1021/acsmaterialsau.4c00064 (PMC11718533; doi:10.1021/acsmaterialsau.4c00064)
Supplement: Supplementary file 1 — mg4c00064_si_001.pdf [file mg4c00064_si_001.pdf]

Supplementary Information

Volumetric passive acoustic mapping and cavitation detection of nanobubbles under low-frequency insonation

Hila Shinar[1] and Tali Ilovitsh[1,2*]

[1] Department of Biomedical Engineering, Tel Aviv University, Tel Aviv 6997801, Israel
[2] The Sagol School of Neuroscience, Tel Aviv University, Tel Aviv 6997801, Israel

* Corresponding author: Tali Ilovitsh
Email: ilovitsh@tauex.tau.ac.il

This PDF file includes:
Supplementary Figures
Fig. S1-S4

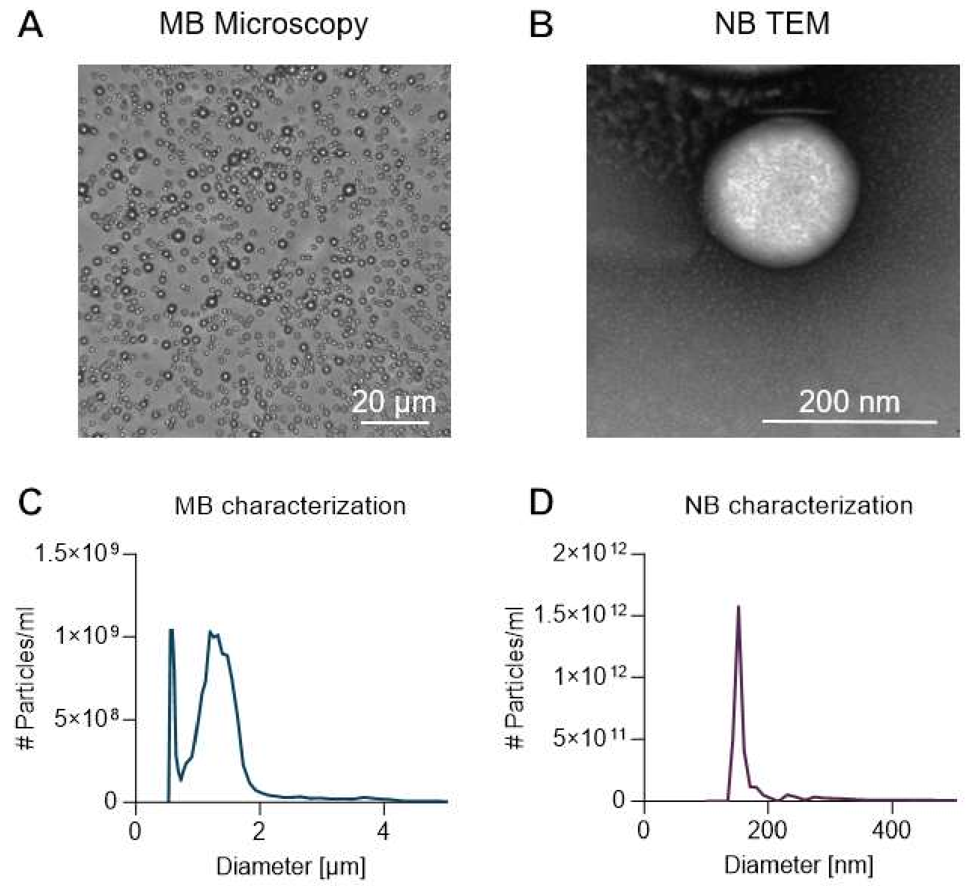

Figure S1. MB and NB characterization.
(**A**) Microscopy image of MB (40x magnification). (**B**) Transmission electron microscopy (TEM) image of NB. (**C**) Size distribution and concentration of MBs, with a median diameter of 1.24 µm. (**D**) Size distribution and concentration of NBs, with a median diameter of 160 nm.

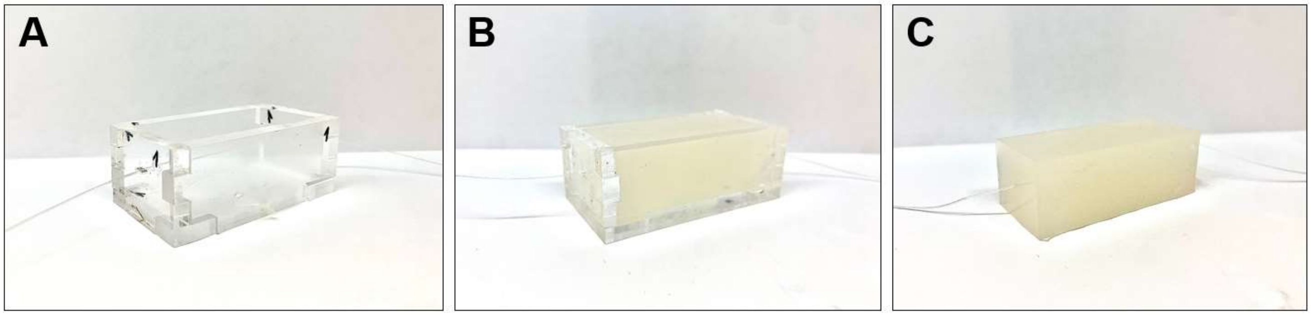

Figure S2. Agarose phantom fabrication.
The preparation process of the agarose phantom. (**A**) The agarose was fabricated in a custom-made laser-cut rectangular mold at a size of 65 mm × 29 mm × 25 mm. (**B**) A solution of agarose powder in deionized water was degassed and poured into the mold to cool down at room temperature until fully solidified. (**C**). Extracted phantom

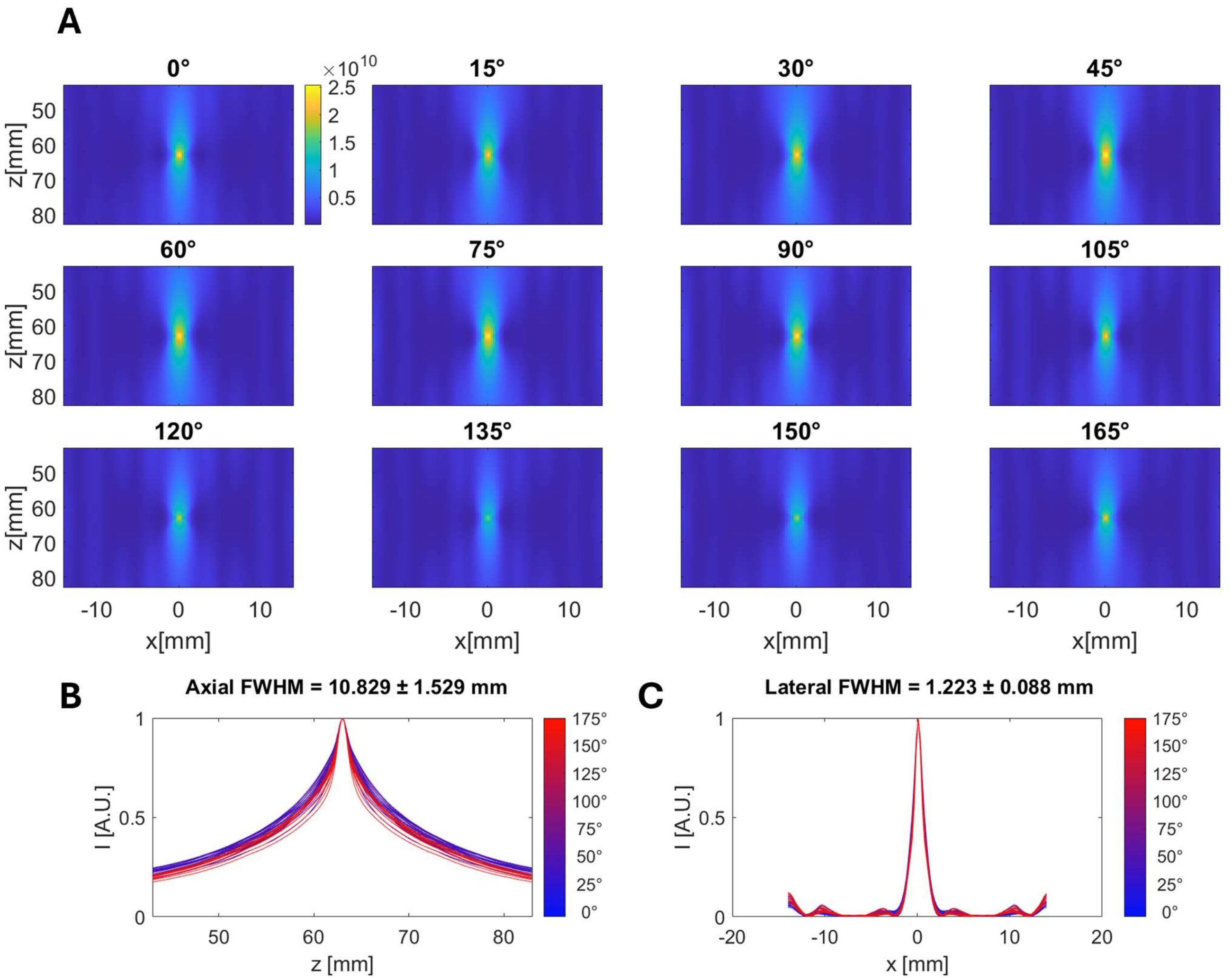

Figure S3. Angle-dependent passive acoustic mapping of microbubbles.
(**A**) Passive acoustic maps of the MBs at different angles. The MBs were excited at 200 kHz and a PNP of 300 kPa. Axes and colorbar are common to all of the subfigures. Colorbar is in A.U. (**B**,**C**) Intensity profiles of the PAM at the axial and lateral directions.

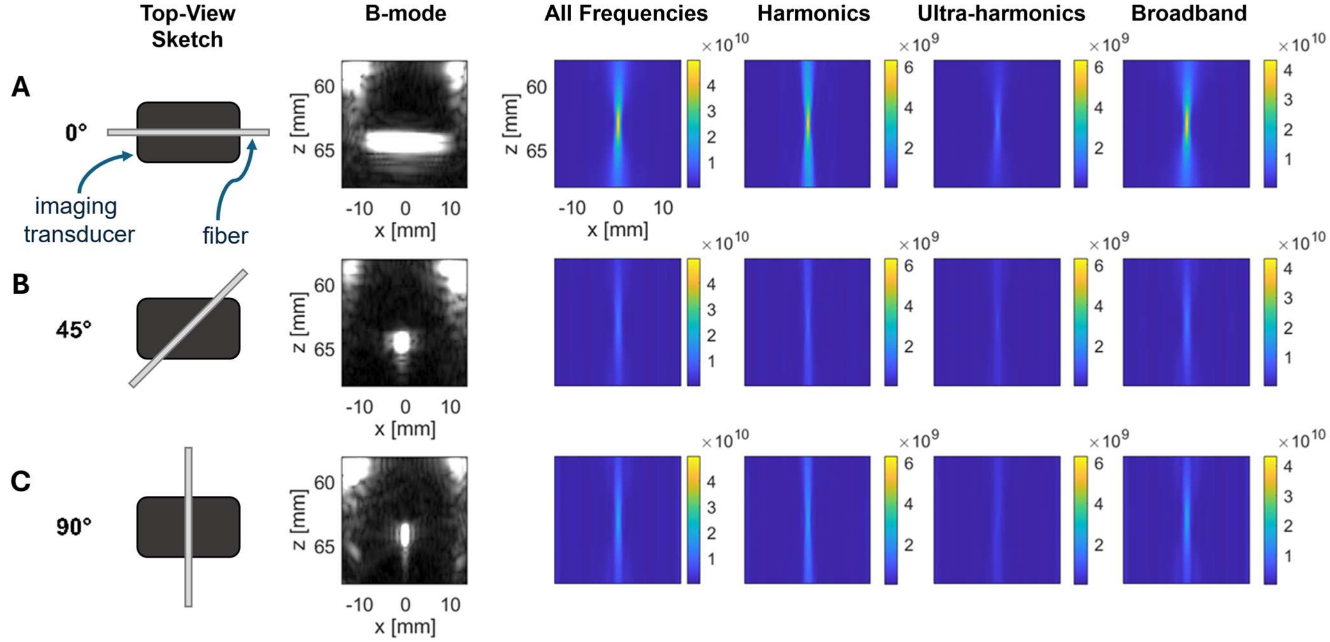

Figure S4. Passive acoustic mapping of microbubbles at 0, 45, 90°.
MBs were excited at 200 kHz and a PNP of 300 kPa. The cavitation activity of the MBs was assessed at (**A**) 0º; (**B**) 45**º**; and (**C**) 90**º**. For each angle, the PAM images are presented, next to the B mode image of the phantom and a top-view sketch demonstrating the orientation of the fiber relative to the imaging transducer. PAM is reconstructed using the entire spectrum (“All Frequencies”), only the harmonics, only the ultra-harmonics, or using the broadband signal. All colorbars are in A.U.
